# Editorial: Unconscious monitoring of physiological information for behavioral changes in daily life: advances in sensor technology and data analysis

**DOI:** 10.3389/fdgth.2025.1697991

**Published:** 2025-10-01

**Authors:** Toshiyo Tamura, Wenxi Chen, Kazuki Nakajima, Shing-Hong Liu

**Affiliations:** ^1^ Future Robotics Organization, Waseda University, Tokyo, Japan; ^2^ Division of Information Systems, School of Computer Science and Engineering, The University of Aizu, Aizuwakamatsu, Japan; ^3^ Division of Bio-information Engineering, University of Toyama, Toyama, Japan; ^4^ Department of Computer Science & Information Engineering, Chaoyang University of Technology, Taichung, Taiwan

**Keywords:** unconscious monitoring, unobtrusive monitoring, non-contact monitoring, biometric authentication, daily activity, assistive technology

**Editorial on the Research Topic**
Unconscious monitoring of physiological information for behavioral changes in daily life: advances in sensor technology and data analysis

Unconscious (non-restrictive) monitoring is an optimal health management method because it does not require participants to wear sensors, cause discomfort, or obtain physiological information with restrictive methods.

This research topic explores sensor advances beyond traditional clinical applications based on clinical examinations. It encompasses the myriad ways that sensors will shape the future of diagnostics, environmental monitoring, and personalized medicine. We aim to capture the essence of the innovations that define the current sensor landscape, from novel sensing techniques to the integration of sensors with digital health platforms.

In the late 1990s, Togawa first proposed such attempts (1), aiming to collect physiological information during sleep, bathing, and toilet use. As the internet became widespread, research in this area was promoted as part of smart home initiatives. However, despite its potential for health management, little clinical evidence was obtained, and further development did not mature enough yet (2).

Additionally, non-contact methods using cameras or radars, as well as smartwatches and wristbands that allow for the collection of various biometric information with minimal constraints, have been incorporated (3, 4).

Recently, AI has been applied to conduct detailed data analyses using various data-driven algorithms for clinical applications. Non-restrictive monitoring, which does not require sensor attachment, has been proven effective in evaluating sleep (5).

This method is also considered an important marker of autonomic nervous system activity (ANSA) and has been proven to predict the likelihood of future health-related events. As data processing techniques have become increasingly important, we have become interested in analyzing the implementation of sensors and biomedical applications. Prolonged camera-based monitoring of critically ill patients presents unique challenges, but it may also facilitate safe recovery. One study was designed to evaluate the feasibility of introducing a non-contact video camera monitoring system into an acute clinical setting (6, 7).

Another possibility is using unobtrusive, wearable equipment to monitor physical activity for wellness, sports, or medical rehabilitation purposes. While many themes overlap, physical rehabilitation, assistive devices, and continuous health monitoring systems are areas of interest (8).

Furthermore, biometric authentication is one example of this application. Facial features and fingerprints are generally used for personal identification. Although still in the research stage, electrocardiogram waveforms can also serve this purpose (9).

This special issue includes six accepted papers. The fields cover two home healthcare devices, two biometric authentication methods, and two assistive devices.

For physiological measurements, Liu et al. derived pulse transit time (PTT) from ballistocardiograms (BCGs) and impedance plethysmograms (IPGs) obtained from a weight-fat scale. Based on these two values, they estimated blood pressure.

Fumimoto et al. propose a method for noncontact measurement of the capillary contraction and dilation which is representative of ANSA using a common commercial camera. They focused on the green-to-blue light ratio during exercise, which is characterized by dominant sympathetic ANS. The G/B ratio decreased during exercise and recovered afterward when the parasympathetic ANS was dominant. Thus, noncontact evaluation of ANS activity was achieved using the G/B ratio.

For biometric authentication, Asano et al. used frequency analysis to label individual spectrograms with a 60 GHz radar and identify the participants.

Meanwhile, Kawamura and Kyoso obtained an electrocardiogram (ECG) from a doorknob, and applied a synchronized averaging to the measured ECG waveforms. After machine learning training, personal identification was realized for security purposes.

Warmerdam et al. hypothesized that there would be characteristic changes in insole-derived vertical ground reaction force (VGRF) and center of pressure (COP) parameters would occur when walking on different surfaces. Analyzing gait data measured via insoles during daily activities further develops physical rehabilitation for the elderly.

Shichitani and Nakajima developed a diaper sensor system to reduce the burden on caregivers. The sensor system detects capacitance changes to quantitatively evaluate the volume of urine absorbed by diapers from different manufacturers with various absorption capacities, and alerts caregivers when a diaper needs to be changed.

Unobtrusive, wearable monitoring could enhance the significance of daily physiological monitoring, even in hospital settings. Further clinical research is needed to popularize this method.
